# Knockdown of Unconventional Myosin ID Expression Induced Morphological Change in Oligodendrocytes

**DOI:** 10.1177/1759091416669609

**Published:** 2016-09-21

**Authors:** Reiji Yamazaki, Tomoko Ishibashi, Hiroko Baba, Yoshihide Yamaguchi

**Affiliations:** 1 Department of Molecular Neurobiology, Tokyo University of Pharmacy and Life Sciences, Hachioji, Tokyo, Japan

**Keywords:** myelin, cytoskeleton, siRNA, myelination

## Abstract

Myelin is a special multilamellar structure involved in various functions in the nervous system. In the central nervous system, the oligodendrocyte (OL) produces myelin and has a unique morphology. OLs have a dynamic membrane sorting system associated with cytoskeletal organization, which aids in the production of myelin. Recently, it was reported that the assembly and disassembly of actin filaments is crucial for myelination. However, the partner myosin molecule which associates with actin filaments during the myelination process has not yet been identified. One candidate myosin is unconventional myosin ID (Myo1d) which is distributed throughout central nervous system myelin; however, its function is still unclear. We report here that Myo1d is expressed during later stages of OL differentiation, together with myelin proteolipid protein (PLP). In addition, Myo1d is distributed at the leading edge of the myelin-like membrane in cultured OL, colocalizing mainly with actin filaments, 2′,3′-cyclic nucleotide phosphodiesterase and partially with PLP. *Myo1d*-knockdown with specific siRNA induces significant morphological changes such as the retraction of processes and degeneration of myelin-like membrane, and finally apoptosis. Furthermore, loss of *Myo1d* by siRNA results in the impairment of intracellular PLP transport. Together, these results suggest that Myo1d may contribute to membrane dynamics either in wrapping or transporting of myelin membrane proteins during formation and maintenance of myelin.

## Introduction

Myelin is a unique multilamellar structure that is crucial for normal neuronal function, including saltatory conduction and maintenance of the neuronal axon. In the central nervous system (CNS), oligodendrocytes (OLs) extend several processes and generate myelin around axons (Nave and Werner, 2014; Bercury and Macklin, 2015). Cytoskeletal organization in OL lineage cells governs cell migration, process extension, myelin wrapping, and myelin compaction. Myelination requires a dynamic membrane transport system using motor proteins. Actin, one of the major cytoskeletal proteins, forms the actin filament (filamentous actin, F-actin) and is involved in intracellular transport near the plasma membrane as well as OL-morphogenesis. Using WAVE-1 knockout mice, researchers demonstrated that actin-related protein (Arp2/3)-dependent actin assembly is required for myelin biogenesis in OL (Kim et al., 2006). Furthermore, both assembly and disassembly of F-actin are crucial for myelin wrapping by OL (Nawaz et al., 2015; Zuchero et al., 2015). However, specific proteins that interact with actin for myelin formation, including membrane trafficking and wrapping, remain to be identified.

The myosin family of cytoskeletal proteins significantly interacts with actin for a variety of cellular functions. To date, two types of myosin have been reported as motor proteins related to the morphological changes in cells of the OL lineage. The first is unconventional myosin VA (Myo5a), a double-headed motor protein that functions in organelle transport. *Myo5a*-null mice exhibit significantly impaired myelination in the brain, optic nerve, and spinal cord (Sloane and Vartanian, 2007). The second is nonmuscle myosin II, which regulates cytoskeletal dynamics in OL progenitors and inhibits OL branching, differentiation, and myelin formation (Wang et al., 2008, 2012). However, because these myosin subfamilies are expressed mainly before forming myelin-like membrane in cultured OLs, these proteins seem to be less involved in the actual myelination process.

Myosin ID (Myo1d), which is an unconventional Class I myosin, is a novel candidate motor protein involved in myelin formation. Myo1d is enriched in the CNS myelin fraction in rat. Subsequent developmental analysis using immunoblotting of the myelin fraction revealed that the Myo1d expression increased beginning 3 weeks after birth, similar to other major myelin proteins (Yamaguchi et al., 2008). Recently, we reported that Myo1d is distributed throughout rat CNS myelin but is especially enriched in the abaxonal and adaxonal regions (the outer and inner cytoplasm-containing loops within the myelin structure, respectively). Myo1d is also expressed in mature OL in culture (Yamazaki et al., 2014). Myo1d (rat: myr4; mouse: myo1g) is composed of a neck containing two IQ calmodulin binding domains and a lipid-binding tail (Sherr et al., 1993; Bähler et al., 1994; Köhler et al., 2005). Although this protein is associated with membrane trafficking along the recycling pathway in Madin-Darby Canine Kidney cells (Huber et al., 2000), its function in OLs remains unclear.

Based on previous findings, we hypothesized that Myo1d is involved in the formation and maintenance of processes and myelin-like-membrane sheets. In this study, to clarify the function of Myo1d in OLs, we examined the timing of the expression of Myo1d and the detailed localization of Myo1d in cultured rat OLs. Finally, we examined the consequences of siRNA-mediated knockdown of *Myo1d* in OLs.

## Materials and Methods

### Animals

Pregnant Wistar rats were purchased from Japan SLC (Hamamatsu, Japan) and maintained in the animal facility of Tokyo University of Pharmacy and Life Sciences. For dissection in preparation of OL primary culture, pregnant Wistar rats were anesthetized using sodium pentobarbital (30–40 mg/kg intraperitoneally; Kyoritsu Seiyaku, Tokyo, Japan). All experiments were conducted in accordance with guidelines on the care and use of animals of Tokyo University of Pharmacy and Life Sciences Animal Use Committee (approval number: P12-19, P13-35, P14-08, P15-25).

### OL Primary Culture

Primary cultures of OL progenitor cells (OPCs) were generated according to the protocol previously described (Yamazaki et al., 2014). OPCs were plated at a density of 4 × 10^4^ cells/well on 13-mm glass coverslips coated with poly-L-lysine in differentiation medium composed of Dulbecco’s modified Eagle’s medium (Wako) with N1 supplement (100 U/ml penicillin, 100 µg/ml streptomycin, 50 µg/ml human apo-transferrin, 10 ng/ml biotin, 25 nM Na selenium, 2.5 µg/ml insulin, 100 µM putrescine, 20 nM progesterone) and 0.5% fetal bovine serum (Gibco/Life Technologies; Stevens et al., 2002). Most of isolated cells were OPCs but slightly included heterogeneously differentiated cells. After differentiation, cells were cultured for 2, 3, 5, or 6 days on coverslips prior to immunostaining.

### Immunofluorescence Staining

Cultured OLs on coverslips were fixed with 4% paraformaldehyde in phosphate-buffered saline (PBS) for 10 min and permeabilized for 5 min at room temperature in 0.1% Triton X-100 in PBS. The coverslips were blocked with Image-iT FX signal enhancer (blocking solution; Molecular Probes, Carlsbad, CA) for 1 hr and then incubated overnight at 4℃ with primary antibodies diluted in blocking solution. After rinsing, the cells were incubated with Alexa Fluor 488 - or 594-conjugated secondary antibodies for 1 hr at room temperature. Finally, the labeled coverslips were rinsed with PBS and mounted onto glass slides with Vectashield containing 4′,6-diamidino-2-phenylindole (DAPI; Vector Laboratories, Burlingame, CA). Images were captured with confocal microscopy (FV100D IX81; Olympus, Tokyo, Japan).

### Developmental Analysis of Myo1d in Cultured OLs

At 2 days after plating on cover slips, OLs were used for immunofluorescence staining. Double immunofluorescence staining was performed using an anti-Myo1d antibody and one of the following anti-OL marker antibodies: anti-O4, anti-myelin basic protein (MBP), or anti-myelin proteolipid protein (PLP) antibodies. The relative ratio of marker-positive cells to the DAPI-positive total cell number in three fields (roughly 30–40 cells/field, 200,000 µm^2^/field) was calculated from three independent cover slips derived from one OL preparation.

### Antibodies for Immunostaining

Specific Myo1d antibody (1:200) was produced by immunization of rabbit with a keyhole limpet hemocyanin conjugated to a 13-aa Myo1d-specific peptide (C-KNRSGFILSVPGN; Yamazaki et al., 2014). The rat hybridoma cell line producing anti-PLP/alternatively spliced isoform of PLP (DM20) monoclonal antibody (AA3; Yamamura et al., 1991; 1:50) was kindly provided by Dr. Kazuhiro Ikenaka (National Institute for Physiological Sciences, Japan). The following antibodies were purchased: rat monoclonal anti-MBP (1:200; Chemicon/Merck Millipore, Billerica, MA), mouse anti-OL marker O4 monoclonal (1:200; Wako Pure Chemical Industries, Osaka, Japan), mouse monoclonal anti-β-tubulin (1:20; Santa Cruz Biotechnology, Dallas, TX), mouse monoclonal anti-2′,3′-cyclic-nucleotide 3′-phosphodiesterase (CNP; 1:100; Sigma-Aldrich Japan, Tokyo, Japan), rabbit polyclonal anti-NG2 proteoglycan (1:200; Chemicon/Merck Millipore), and rabbit polyclonal anti-caspase3 (1:100; Cell Signaling Technology, Danvers, MA). The secondary antibodies used for immunostaining were Alexa Fluor 488 - and 594-conjugated species-specific antibodies (1:2000; Molecular Probes/Life Technologies).

### siRNA Treatment

Three sets of double-strand siRNA oligonucleotides against the rat *Myo1d* gene and a set of universal negative control-siRNA were purchased from Sigma-Aldrich Japan. OLs cultured in differentiation medium at 72 hr or 120 hr after plating were transfected with 100 nM fluorescence-labeled-siRNA or unlabeled-siRNA against *Myo1d*- or with control-siRNA for either 48 hr (at 3 days after differentiation) or 24 hr (at 5 days after differentiation) with the MISSION siRNA Transfection Reagent (Sigma-Aldrich Japan) according to the manufacturer’s protocol. Since spontaneously damaged cells are increased from 6 days in differentiation medium, siRNA treatment at 5 days after differentiation is only for 24 hr. According to the results of RT-PCR analyses of cultured OLs using three sets of *Myo1d-*siRNA (data not shown), we selected one of the most effective *Myo1d-*siRNAs. The sequences of the double strand *Myo1d*-siRNA set were as follows:

Sense siRNA: 5′-GAAUCGAUUUAGUAAGGUATT-3′

Antisense siRNA: 5′-UACCUUACUAAAUCGAUUCTT-3′

For quantitative analysis in siRNA experiments, the relative ratio of transfected cells to the DAPI-positive total cell number in three fields (roughly 30–40 cells/field; 3.969 × 10^5 ^µm^2^/field) was calculated from independently treated cover slips derived from two or three OL preparations.

### RT-PCR Analysis

Total RNA was extracted from primary cultured OLs grown in six-well tissue culture plate (8 × 10^4^ cells/well) using a TRIzol Plus RNA Purification Kit (Invitrogen/Life Technologies). The isolated total RNA was then reverse transcribed with a TaKaRa RNA LA Kit (AMV) Ver. 1.1 (Takara Bio, Shiga, Japan). *Myo1d* and control glyceraldehyde-3-phosphate dehydrogenase (*Gapdh*) cDNA were amplified by PCR with the following specific primer sets:

*Myo1d* forward; 5′-TGCTGACGCTGCTTACAAGGC-3′

*Myo1d* reverse; 5′-TCTATCTCTGCTCTCTGGCTG-3′

*Gapdh* forward; 5′-AATGGTGAAGGTCGGTGTGAAC-3′

*Gapdh* reverse; 5′-GAAGATGGTGATGGGCTTCC-3′

Amplified products were analyzed using an agarose electrophoresis gel stained with ethidium bromide. For quantification, the intensity of each band was measured by ImageGauge v4.23 (Fujifilm, Tokyo, Japan) and the relative ratio of *Myo1d* to *Gapdh* was calculated. Values were obtained from three OL preparations.

### Time-Lapse Imaging Using Confocal Microscopy

Three hours after siRNA transfection, OLs were labeled with calcein AM solution (1:6000; Dojindo, Kumamoto, Japan). Calcein-labeled OLs were cultured for 24 hr in siRNA-containing differentiation medium. Time-lapse images were captured every hour from 4 hr to 24 hr after transfection using confocal microscopy (FV1000-D IX81; Olympus). Videos were reconstructed from the captured images of 4 hr to 12 hr after transfection (Supplemental Videos). For quantitative analysis, the relative ratio of calcein-positive cells to total cells was calculated for each of five fields (roughly 30–40 cells/field; 3.969 × 10^5 ^µm^2^/field) at 6 hr after transfection was calculated from each coverslip for each of two OL preparations.

### Statistical Analysis

All of the quantitative analyses are presented as the mean ± standard error of the mean (*SEM*) from three experiments. The statistical analyses were performed using Prism 5 (GraphPad Software, La Jolla, CA). The statistical comparisons were performed by Student’s *t*-test or one-way ANOVA, followed by Turkey Kramer test. The level of significance was indicated by *p* value: *p < .05 and **p < .01.

## Results

In this study, we used rat primary OL culture to analyze the role of Myo1d on OL differentiation and myelination. The differentiation observed in primary culture OL corresponds closely to the developmental process *in vivo*. In the primary culture system, the OL differentiation process proceeds through four discrete stages: an OPC, the pre-OL, the immature OL, and finally the mature, myelinating OL (Pfeiffer et al., 1993; Baumann and Pham-Dinh, 2001; Barateiro and Fernandes, 2014). Morphologically bipolar OPCs can be identified by the expression of NG2 chondroitin sulfate proteoglycan. NG2 is detected until the pre-OL stage while the cell surface marker, O4-antigen, is expressed beginning at the pre-OL stage onward. During the pre-OL and immature OL stages, OLs increase the number of processes and extends each process. When OLs become mature, each OL expresses MBP first and PLP in a little later stage to form myelin-like membrane.

### Myo1d is Expressed in Mature OLs but is not Detected at Earlier Stages of Differentiation

**Figure 1.**
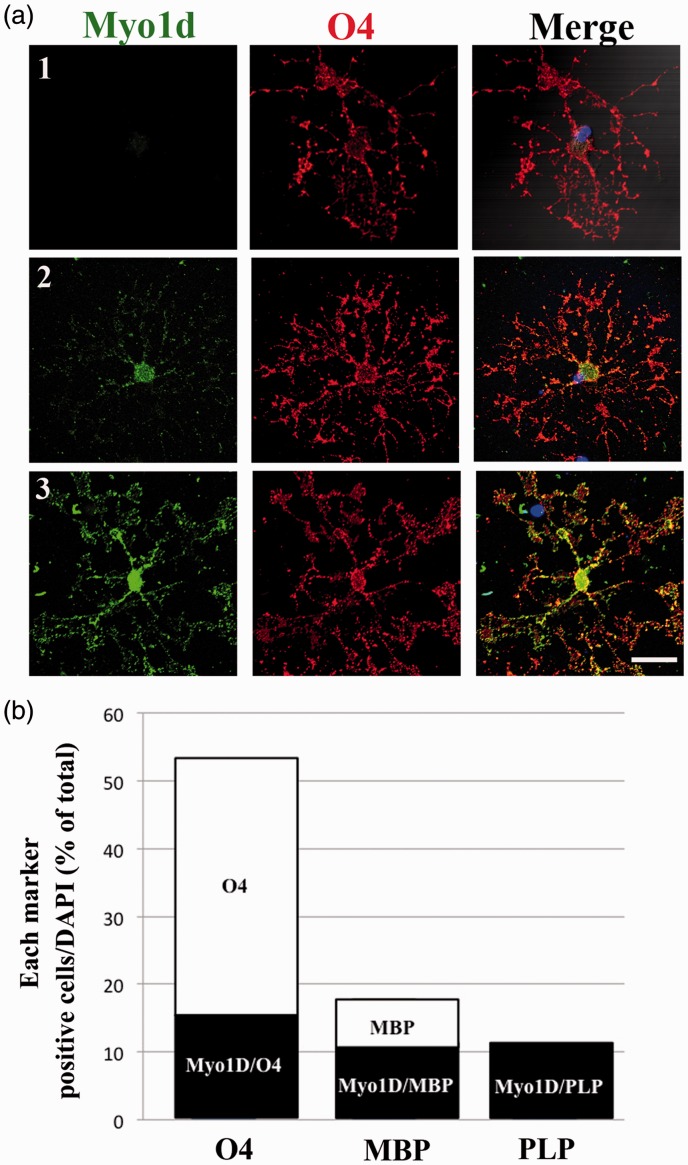
Timing of Myo1d expression during the differentiation of cultured oligodendrocytes (OLs). (a) After 2 days in differentiation medium, OLs were double stained with anti-Myo1d (green) and anti-O4 (red) antibodies. Three types of representative O4-positive OLs are shown: O4-positive, Myo1d-negative OLs (a1), O4-positive OLs in which Myo1d-positive signal was detected primarily in the cell body but not in processes (a2), and O4-positive OLs in which Myo1d-positive signal was detected in cell body, processes, and the leading edge (a3). Nuclei were counterstained with DAPI (blue). Scale Bar, 20 µm. (b) Double immunofluorescent staining of cultured OLs at 2 days after plating using anti-Myo1d antibody and either anti-O4, anti-MBP, or anti-PLP antibodies. Percent of each marker-positive cells as compared with total DAPI-positive cells was calculated from three cover slips derived from one OL preparation (*n* = 3). Percentages are 29.2%, 62.1%, and 100%, respectively.

As OL develop toward maturity, OLs begin expressing myelin proteins such as MBP and PLP. During brain development, similar to MBP, the expression of Myo1d increases with the maturation of white matter of cerebellum and corpus callosum; Myo1d is also expressed in PLP-positive mature OLs cultured *in vitro* (Yamazaki et al., 2014). However, the timing of *Myo1d* expression during OL differentiation stage is still unknown. To determine when Myo1d was expressed during the OL differentiation process, double immunofluorescence staining of cultured OL was performed after 2 days in differentiation medium. At this time, while most of the cells had differentiated to O4-positive cells, their morphology indicated their differentiation was heterogeneous, including both immature and mature OLs. Many O4-positive OLs, that had fewer processes, were Myo1d-negative (Figure 1(a1)). Myo1d-positive signals were only detected in O4-positive OLs that had many processes, either with (Figure 1(a3)) or without (Figure 1(a2)) myelin-like membrane. This result suggests that Myo1d is expressed in mature OLs but not in immature OLs (O4^+^, MBP^−^, PLP^−^). Next, to determine the detailed timing of Myo1d expression, we calculated percent of cells expressing each marker as compared with total DAPI-positive cells using immunofluorescence after two days in differentiation medium. The percentages of O4^+^, MBP^+^, and PLP^+^ cells in the total DAPI-positive population were 53.6%, 18.5%, and 10.5%, respectively. Furthermore, the percentages of Myo1d-positive cells in the O4^+^, MBP^+^, or PLP^+^ cell populations were 29.2%, 62.1%, and 100%, respectively (Figure 1(b)). Thus, the timing of Myo1d expression coincides with that of PLP in cultured OLs.

### Myo1d Colocalizes With F-Actin at the Leading Edge of the Myelin-Like Membrane on Mature OLs

**Figure 2.**
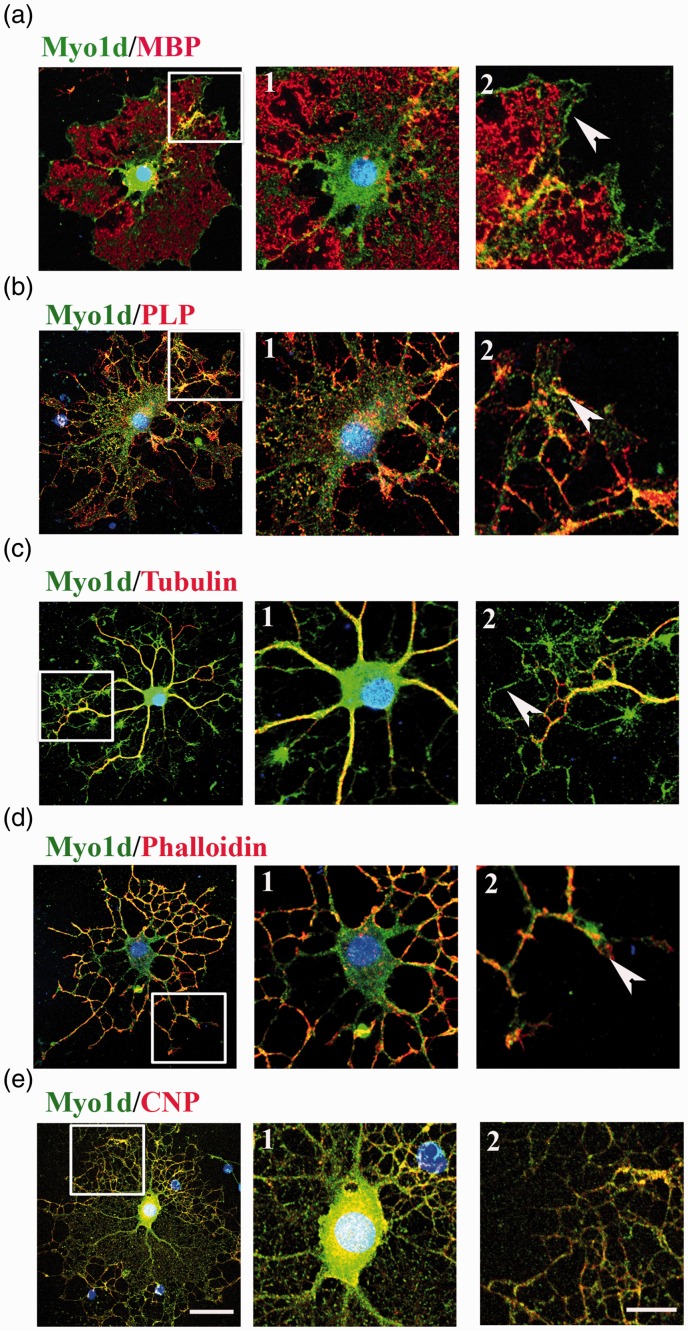
Localization of Myo1d in cultured OL. Double immunofluorescent staining of Myo1d (green) with (a) MBP, (b) PLP, (c) tubulin, (d) F-actin (phalloidin), or (e) CNP (red) was performed in mature OLs (a1–e1, enlargement of cell body; a2–e2, enlargement of distal part of process indicated by white square in left panel). Nuclei were counterstained with DAPI (blue). Myo1d-positive signals were enriched at the leading edge of MBP-positive myelin-like membrane sheets (a2). Many PLP- and CNP-positive signals were colocalized with Myo1d-positive signals (b2 and e2). Phalloidin signals were colocalized with Myo1d signals in distal, thinner processes (d1) especially at the tips of processes (a2–d2, arrowheads). Scale bars: 50 µm in (e), applies to (a) to (e), left panels; 20 µm in (e2), applies to (a1) to (e2).

To examine the detailed distribution of Myo1d in OLs, we performed double immunofluorescence analysis of cultured OL using anti-MBP, anti-PLP, anti-tubulin, anti-CNP, and anti-Myo1d antibodies and fluorescence-labeled phalloidin. Five days after plating on coverslips in differentiation medium, many of the OLs became mature. Punctate staining of Myo1d was distributed throughout the cell body, on the thicker processes extending from the cell body, and on the leading edge of the myelin-like membrane sheet; yet, Myo1d was not observed in MBP-positive myelin-like membrane sheets (Figure 2(a)). Many of the Myo1d-positive signals were colocalized with PLP-positive signals (Figure 2(b)), especially in the cell body (Figure 2(b1)) and processes (Figure 2(b2)). Myo1d signals were also colocalized with tubulin signals in the proximal, thicker processes (Figure 2(c)); however, tubulin signals were not found in distal, thinner processes nor on the leading edges of the myelin structure (Figure 2(c2)). Staining with fluorescent phalloidin revealed that Myo1d signals were colocalized with actin filaments in the distal, thinner processes and on the leading edges (Figure 2(d)). CNP-positive signals significantly overlapped with Myo1d-positive signals in many places (Figure 2(e)). These results indicate that Myo1d is closely localized with actin filaments and CNP and partially localized with PLP.

### Knockdown of *Myo1d* Expression Induces Morphological Changes in OLs

**Figure 3.**
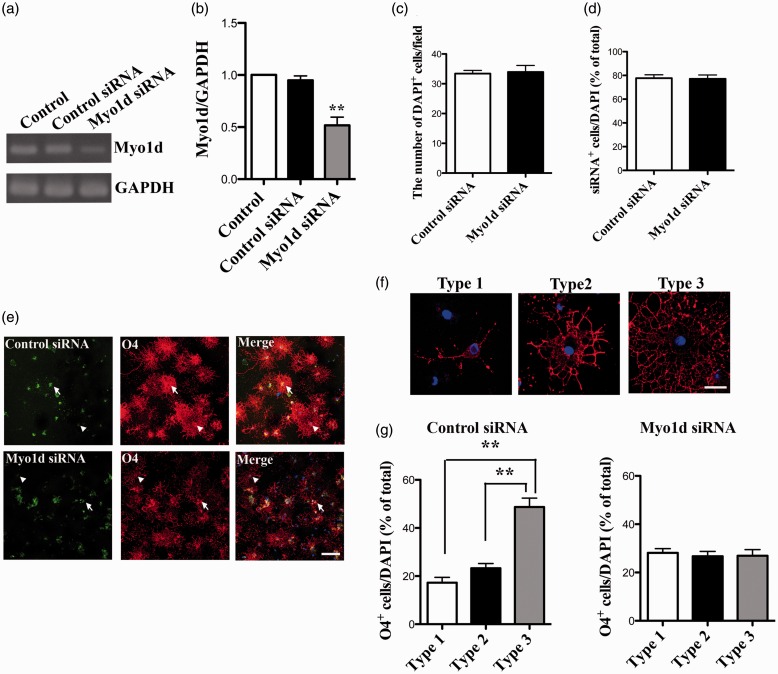
Knockdown of *Myo1d* in cultured OLs using siRNA. (a) Cultured OLs were transfected with *Myo1d*- or control-siRNA (100 nM each) for 48 hr beginning 3 days after plating. Nontransfected (Control) and OLs transfected with *Myo1d*- or control-siRNA were examined by RT-PCR analysis for *Myo1d* (above) and *Gapdh* (below) mRNA expression. (b) For quantitative analysis, each band was normalized to the *Gapdh* band. Graphs indicate the mean ± *SEM* obtained from three independent OL preparations (*n* = 3). Asterisks indicate *p* < .01 by Tukey-Kramer test after one-way ANOVA. (c, d) The numbers of DAPI-positive total cells (c) and the transfection efficiencies (d) were not different between control- and *Myo1d*-siRNA-treated cells. Graphs indicate the mean ± *SEM* obtained from three independent OL preparations (*n* = 3). For quantitative analysis, a Student’s *t*-test was performed. (e) To confirm the transfection of siRNA in cultured OL, cells were transfected with fluorescence-labeled *Myo1d*-siRNA or fluorescence-labeled control-siRNA for 48 hr. After transfection, we performed immunostaining using anti-O4 antibody (red). Transfected cells were detected by fluorescence-labeled-siRNA (green). Arrow, siRNA-positive cells; Arrowhead, siRNA-negative cells. Scale bar, 100 µm. (f) OLs were classified into three groups according to anti-O4-stained (red) morphology, representative images of O4-stained OL of each group are shown. Type 1: little to no branching; Type 2: some branching, with processes < 30 µm and MBP localized only to the cell body; Type 3: extensive branching, with processes > 30 µm and MBP localized in both cell body and processes. Scale bar, 20 µm. (g) Comparison of the percent of each type of OLs as compared with total DAPI-positive cells in control- and *Myo1d*-siRNA-treated OLs. Type 1 OLs were increased by *Myo1d*-siRNA-transfection, while Type 3 OLs were decreased. Graphs indicate the mean ± *SEM* obtained from three independently treated derived from three OL preparations (*n* = 9). Asterisks indicate *p* < .01 by Tukey-Kramer test after one-way ANOVA. Nuclei were counterstained with DAPI (blue).

To clarify the function of Myo1d in OLs, we performed siRNA-mediated knockdown of *Myo1d* in cultured OLs after three days in differentiation medium. OLs were transfected with *Myo1d*- or control-siRNA (100 nM each) for 48 hr. In RT-PCR analysis, while the expression of *Gapdh* mRNA was unchanged, *Myo1d* mRNA expression was efficiently suppressed by siRNA treatment (Figure 3(a)). When quantified with respect to *Gapdh* mRNA, *Myo1d* expression in cultured OLs was significantly reduced in *Myo1d*-siRNA-treated samples as compared with control-siRNA-treated samples (Figure 3(b); control, 1.0; control siRNA, 0.95 ± 0.042; Myo1d siRNA, 0.52 ± 0.078; *p* < .01). The number of total DAPI-positive cells did not change after siRNA transfection (Figure 3(c); control siRNA, 34.4 ± 1.1 cells; Myo1d siRNA, 33.9 ± 2.2 cells). Transfection using fluorescence-labeled siRNA revealed that approximately 80% of OLs were transfected by siRNA; the transfection efficiency was similar for control- and *Myo1d*-siRNA (Figure 3(d); control siRNA, 78.7 ± 2.9%; Myo1d siRNA, 78.2 ± 3.2%). Using immunofluorescence staining with anti-O4 antibody (red), morphological changes were observed in *Myo1d*-siRNA (green)-transfected OLs but not in untransfected or control-siRNA (green)-transfected OLs (Figure 3(e)).

To analyze OL morphological changes observed after *Myo1d*-siRNA treatment, OLs were classified into three different groups according to the specific morphology visualized by O4 staining (Figure 3(f)): Type 1, low complexity with only a few processes; Type 2, medium complexity with several branches of processes extending up to 30 -µm from the cell body; and Type 3, high complexity with many branches of processes spread more than 30 µm from the cell body. For quantitative analysis, the percent of each type of OL to total DAPI-positive cells was calculated. While the ratio of Type 3 OLs was significantly higher in control-siRNA-transfected OLs (Figure 3(g) left, control siRNA; Type 1, 17.3 ± 2.2%; Type 2, 23.3 ± 1.9%; Type 3, 48.7 ± 3.7%; *p* < .01), there was no difference among the percentages of each of the morphological types in *Myo1d*-siRNA-transfected OLs (Figure 3(g) right, Myo1d siRNA; Type 1, 28.1 ± 1.8%; Type 2, 26.7 ± 2.0%; Type 3, 26.9 ± 2.6%). These results indicate that *Myo1d*-specific siRNA-treatment efficiently reduced the expression of *Myo1d* mRNA in OLs and induced the inhibition of extension or degeneration of processes in OLs.

### *Myo1d* Knockdown Decreases the Number of MBP-Positive Cells and May Cause Degeneration of Myelin-Like Membrane

**Figure 4.**
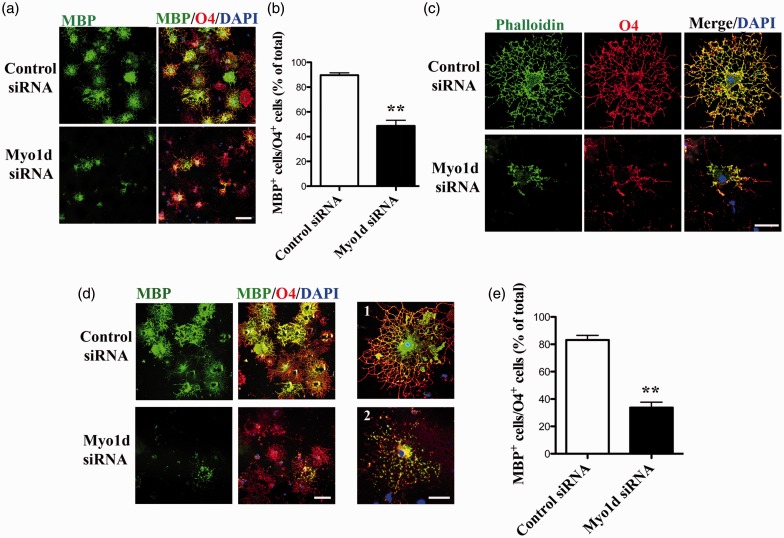
Decrease in the number of mature OL after knockdown of *Myo1d* expression. (a) Cultured OLs were transfected with *Myo1d*- or control-siRNA (100 nM each) for 48 hr beginning 3 days after differentiation. *Myo1d*-siRNA-transfected OLs were double-stained using anti-MBP (green) and anti-O4 antibodies (red). Scale bar, 100 µm. (b) The number of MBP-positive OLs was significantly decreased after *Myo1d* siRNA transfection. Graphs indicate the mean ± *SEM* obtained from three independently treated cover slips derived from three OL preparations (*n* = 9). Asterisks indicate *p* < .01 by Student’s *t*-test. (c) *Myo1d*-knockdown OLs were stained using fluorescence-labeled phalloidin (green) and anti-O4 antibody (red). Cytoskeletal F-actin was disrupted in *Myo1d*-siRNA-transfected OL (lower panel). Scale bar, 50 µm. (d) Cultured OLs were transfected with *Myo1d*- or control-siRNA (100 nM each) for 24 hr beginning 5 days after differentiation. OLs were double stained using anti-MBP (green) and anti-O4 antibodies (red). Scale bar, 100 µm in (d). Representative control-siRNA- (d1) or *Myo1d*-siRNA-transfected OLs (d2) were exhibited. Scale bar, 50 µm in (d2). (e) In quantitative analysis, the ratio of membrane-positive OLs (i.e., MBP-positive membrane sheets-positive OLs) to O4-positive OLs were significantly decreased by *Myo1d*-siRNA transfection. Graphs indicate the mean ± *SEM* obtained from three independently treated cover slips in three OL preparations (*n* = 9). Asterisks indicate *p* < .01 by Student’s *t*-test. Nuclei were counterstained with DAPI (blue) staining.

To investigate the maturation level of *Myo1d*-siRNA-transfected OLs, we performed double immunostaining using anti-MBP and anti-O4 antibodies (Figure 4(a)). In quantitative analysis, the percentage of MBP-positive mature OLs was significantly decreased after *Myo1d* siRNA transfection (Figure 4(b); control siRNA, 91.8 ± 1.9%; Myo1d siRNA, 45.1 ± 5.6%; *p* < .01). In addition, MBP-positive membrane sheets were nearly undetectable in MBP-positive mature OLs after treatment with *Myo1d*-siRNA (Figure 4(a), lower panel). Similar results were observed in independent *Myo1d*-knockdown experiments using two other *Myo1d*-siRNA sets (Supplemental Figure). These results indicate that Myo1d may be involved in the formation of myelin-like membrane sheets.

To examine cytoskeletal changes in *Myo1d*-knockdown cells, OLs were stained with fluorescence-labeled phalloidin (Figure 4(c)). After *Myo1d*-siRNA treatment, many O4-positive cells exhibited degenerated processes. These data suggest that morphological changes in *Myo1d*-siRNA-transfected cells are associated with the disruption of cytoskeletal actin filaments.

Furthermore, we investigated whether degeneration of myelin-like membrane could be induced by *Myo1d*-siRNA-transfection in highly differentiated OLs. After 5 days in differentiation medium, when most of cells became highly differentiated OLs (Type 3 OLs in Figure 3), approximately 80% of OLs were MBP-positive (data not shown). Then, all OLs were transfected with *Myo1d*- or control-siRNA (100 nM each) for 24 hr. After fixation, cells were double stained with anti-MBP and anti-O4 antibodies (Figure 4(d)). MBP-positive myelin-like membrane sheets were observed in control OL (Figure 4(d1)), yet were severely disrupted and almost undetectable after knockdown of *Myo1d*-expression (Figure 4(d2)). The percentage of OLs containing MBP-positive membrane sheets was significantly decreased after *Myo1d*-siRNA transfection (Figure 4(e); control-siRNA, 82.9 ± 2.8%; Myo1d-siRNA, 33.5 ± 4.4%; *p* < .01). These results indicate that Myo1d may be required for the maintenance of myelin-like membrane sheets.

### Knockdown of *Myo1d* Expression May Cause Inhibition of Differentiation and Induction of Apoptosis in OLs

**Figure 5.**
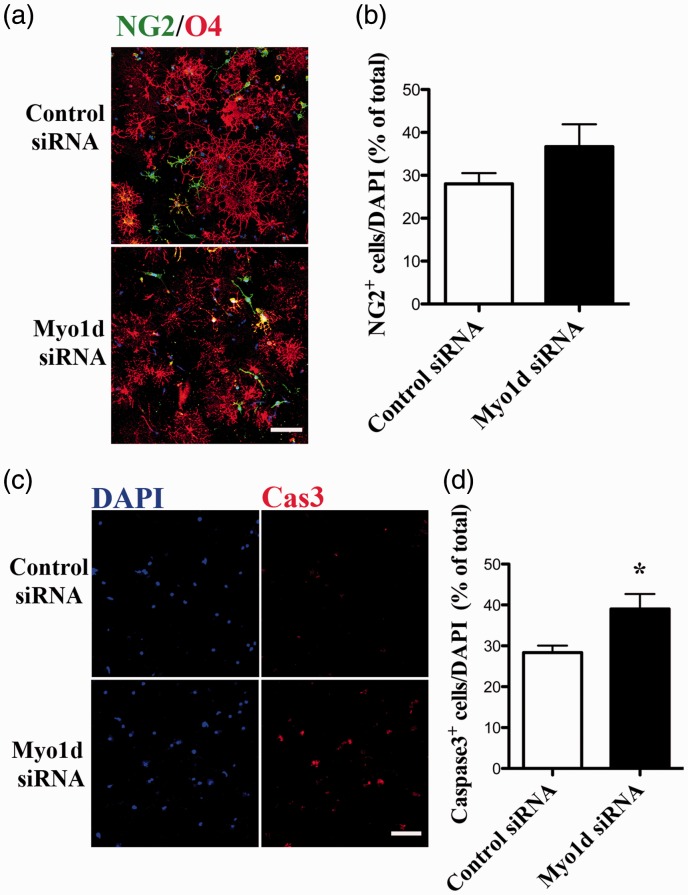
Analysis of inhibition of differentiation and apoptosis of *Myo1d*-siRNA-transfectrd OLs. (a) Cultured OLs were transfected with *Myo1d*- or control-siRNA (100 nM each) for 48 hr beginning 3 days after differentiation. siRNA-transfected OLs were stained with anti-NG2 (green) and anti-O4 (red). Scale bar, 100 µm. (b) In quantitative analysis, the ratio of NG2-positive cells tended to increase after Myo1d-siRNA transfection. (c) siRNA-transfected OLs were stained using anti-caspase3 (red). Scale bar, 100 µm. (d) The ratio of caspase3 positive cells to DAPI-positive total cells increased after *Myo1d*-siRNA transfection. Graphs indicate the mean ± *SEM* obtained from two independently treated cover slips derived from three OL preparations (*n* = 6). Asterisks indicate *p* < .05 by Student’s *t*-test. Nuclei were counterstained with DAPI (blue).

To investigate the influence of *Myo1d* knockdown treatment on the level of differentiation, 3 days after differentiation, we performed double immunostaining using anti-NG2 (OL-progenitor marker) and anti-O4 (Figure 5(a)) antibodies. NG2-positive cells were detected among morphologically changed OLs after *Myo1d*-siRNA treatment. The percentage of NG2-positive cells tended to be increased after Myo1d-siRNA transfection (Figure 5(b); control-siRNA, 28.5 ± 2.4%; Myo1d-siRNA, 38.1 ± 4.8%). This result suggests that inhibition of differentiation may be caused by knockdown of *Myo1d* expression in cultured OLs.

The degeneration of processes and the stalls of transports of myelin proteins in OLs may induce apoptosis. Therefore, next, to determine whether *Myo1d*-knockdown affects apoptosis of OLs, we stained OLs with anti-caspase3 antibody (Figure 5(c)). Strong caspase3-positive staining was observed in *Myo1d*-siRNA treated OLs, but only background fluorescence staining was observed in control OLs. The percentage of caspase3-positive cells significantly increased after *Myo1d*-siRNA-transfection (Figure 5(d); control-siRNA, 29.5 ± 3.1%; *Myo1d*-siRNA, 38.7 ± 4.2%; *p* < .01). These results suggest that knockdown of *Myo1d* expression partly induced apoptosis in cultured OL.

### *Myo1d* Knockdown May Induce the Retraction of Processes in OLs

**Figure 6.**
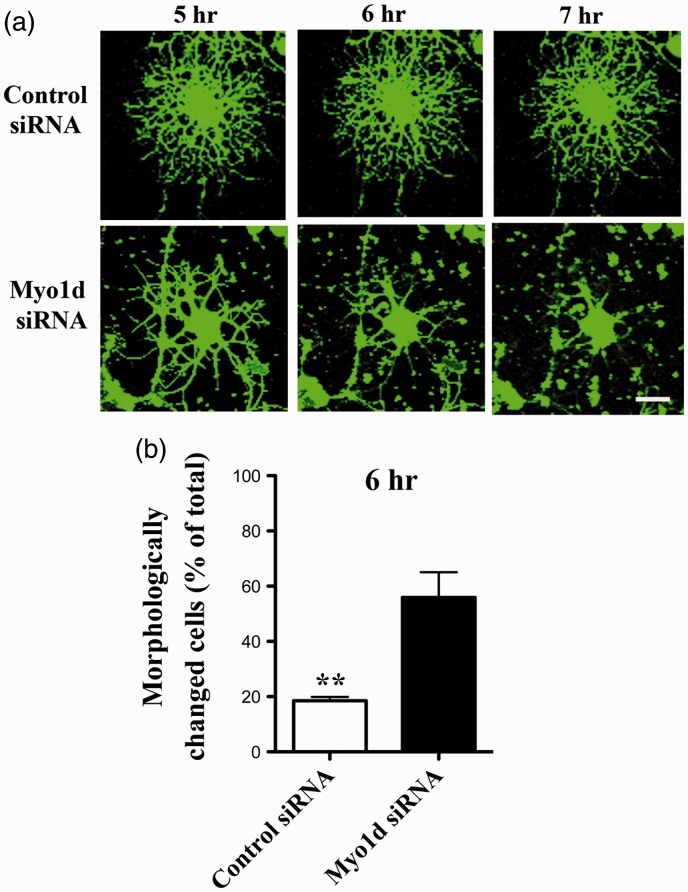
Time-lapse analysis of cultured OL by siRNA knockdown of *Myo1d*. (a) At 3 hr after siRNA transfection, OLs were labeled using fluorescent dye (calcein AM). Fluorescent images were captured at 5, 6, and 7 hr after transfection. OLs started to retract their processes beginning 6 hr after *Myo1d*-siRNA transfection (arrow). Scale bar, 20 µm. (b) The percentage of OLs whose processes were retracted after *Myo1d*-siRNA transfection was calculated with respect to total fluorescent-labeled cells. Graph indicates the mean ± *SEM* obtained from each of 5 fields at 6 hr after transfection derived from two OL preparations (*n* = 10). Asterisks indicate *p* < .01 by Student’s *t*-test. Nuclei were counterstained with DAPI (blue).

To further investigate the process of morphological changes of *Myo1d*-siRNA-transfected OLs, we performed time-lapse analysis of differentiated OLs transfected with *Myo1d*-siRNA at three days after differentiation. Cells were visualized using calcein AM. Time-lapse analysis of siRNA-transfected OLs revealed the retraction of processes. Retraction was found in many cells in 6 hr after *Myo1d*-siRNA transfection (Figure 6(a); Supplemental Videos). Quantitatively, the ratio of process-retracted OLs to fluorescence-labeled total cells was significantly increased after *Myo1d*-siRNA treatment (Figure 6(b); control-siRNA; 18.5 ± 2.9%, *Myo1d*-siRNA; 56.0 ± 11.2%; *p* < .01). Therefore, Myo1d may be involved in the maintenance of myelin in cultured OL.

### Knockdown of *Myo1d* Expression May Impair PLP Transport in Cultured OLs

**Figure 7.**
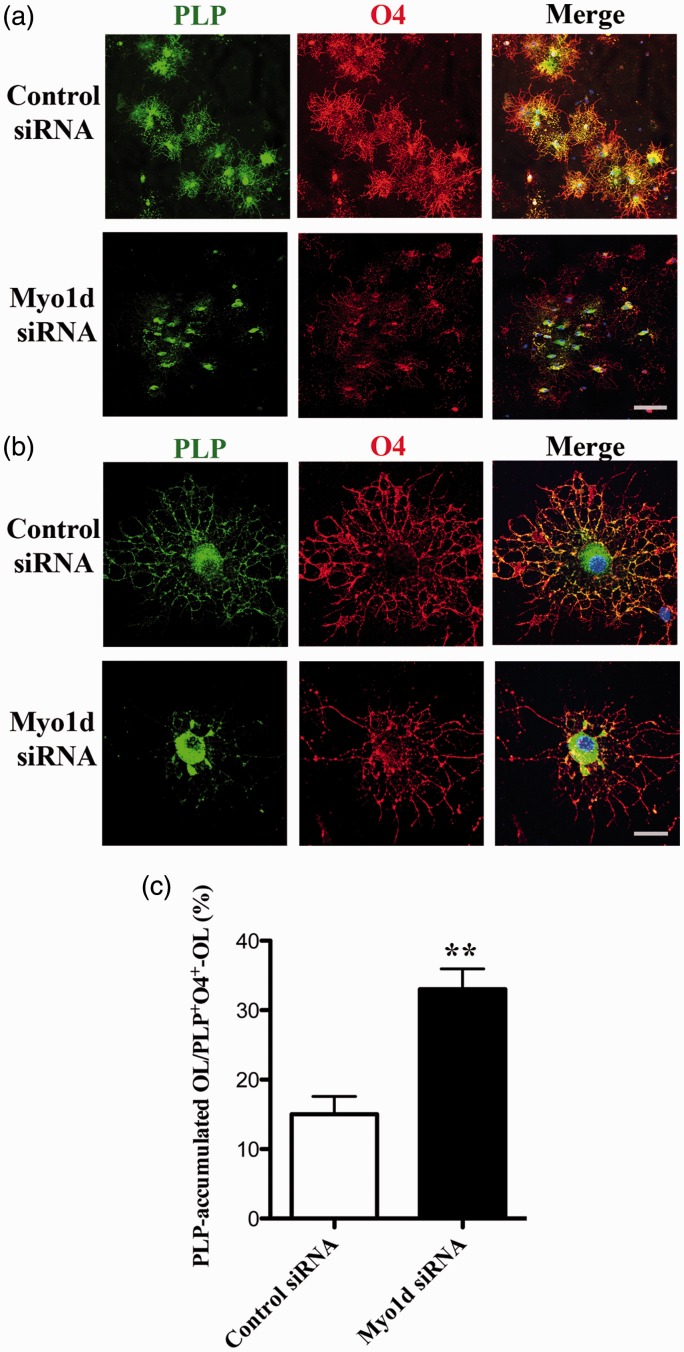
Analysis of impairment of PLP transport by *Myo1d*-specific siRNA transfection. (a) Cultured OLs were transfected with *Myo1d*-siRNA or control-siRNA (100 nM each) for 48 hr beginning 3 days after differentiation. siRNA-transfected OLs were stained with anti-PLP (green) and anti-O4 (red) antibodies. PLP-positive signals were detected in the cell body after *Myo1d*-siRNA transfection (arrowhead). Scale bar, 100 µm. (b) Enlargements of morphologically representative cells in control-siRNA (upper) or *Myo1d*-siRNA (lower)-transfected OLs are exhibited. PLP-positive-signal accumulated in the cell bodies of *Myo1d* siRNA transfected cells. Scale bar, 50 µm. (c) The percentage of OLs in which PLP-positive signals accumulated in cell body but not the distal processes as compared with total PLP- and O4-positive OLs was significantly increased after *Myo1d*-siRNA transfection. Graph indicates the mean ± *SEM* obtained from three independently treated cover slips derived from two OL preparations (*n* = 6). Asterisks indicate *p* < .01 by Student’s *t*-test. Nuclei were counterstained with DAPI (blue).

The timing of the expression of Myo1d in cultured OL coincides with that of PLP, and Myo1d-positive signals were partially colocalized with PLP-positive signals. To investigate whether knockdown of *Myo1d* expression affected PLP transport, we performed siRNA-mediated knockdown of *Myo1d* in cultured OLs at 3 days after differentiation. OLs were treated with *Myo1d*- or control-siRNA (100 nM each) for 48 hr. After siRNA treatment, cells were double stained with anti-PLP and O4 antibodies (Figure 7(a) and (b)). In control-siRNA-transfected OLs, most cells exhibited PLP expression in cell bodies as well as in processes (Figure 7(a) and (b); upper panels). However, in *Myo1d*-specific siRNA-transfected OLs, PLP-positive signals were primarily found in cell bodies while most O4-positive processes were PLP-negative (Figure 7(a) and (b); lower panels). The percentage of restrictedly cell body, PLP-positive and O4-positive OLs to total O4-positive OLs was significantly increased by transfection with *Myo1d*-siRNA (Figure 7(c); control siRNA, 15.0 ± 2.6; *Myo1d* siRNA, 33.0 ± 3.0; *p* < .01). The processes of these cells were O4-positive but PLP-negative. From these results, we hypothesized that Myo1d may be involved in PLP transport during myelination.

## Discussion

Myo1d is an unconventional myosin expressed in OL, whose function remains unclear. In this study, we demonstrated that Myo1d expression begins at the same time as PLP during the differentiation of cultured, mature OL. Localization analysis of Myo1d in cultured OLs indicated that Myo1d was enriched at the leading edge of the myelin-like membrane, colocalizing primarily with actin filaments, CNP, and sometimes with PLP. *Myo1d* siRNA knockdown revealed that the loss of *Myo1d* induced morphological changes (especially retraction of processes and degeneration of the myelin-like membrane) resulting in apoptosis and impairment of PLP transport in cultured OL. We, therefore, propose that Myo1d contributes to myelin membrane dynamics in OL.

Myosin family members interact with actin filaments to drive motors to maintain cell shape or to form myelin. Nonmuscle myosin II and Myo5a have been reported as myosins expressed in OL lineage cells (Sloane and Vartanian, 2007; Wang et al., 2008, 2012; Rusielewicz, 2014). Nonmuscle myosin II functions as a negative regulator of differentiation and myelination of OL; therefore, loss of nonmuscle myosin II accelerates repair of demyelinated lesions in the corpus callosum (Wang et al., 2008, 2012; Rusielewicz, 2014). Myo5a, associated with vesicle-associated membrane protein-2 (VAMP2), is involved in morphogenesis and myelination of OL in mice (Sloane and Vartanian, 2007). These myosins are primarily involved in morphological function during early stages of OL differentiation. In contrast, Myo1d is expressed with PLP during later stages of OL differentiation, possibly to be involved in myelin formation. Thus, to switch functions during morphogenesis, each myosin seems to be expressed according to a distinct timetable.

Formation and wrapping of the myelin membrane are the principle events during the later stages of OL development. OL extends many processes; then, after individually attaching to an axon, OL wraps its own plasma membrane around the process to form multilamellar myelin. During the wrapping process, the myelin membrane grows unidirectionally at the leading edge of innermost layer (inner tongue). The innermost layers of myelin contain cytoplasm and are connected to outermost edge and the cell body by a channel for transporting proteins and membranes (Snaidero et al., 2014). Initial process extension by OL requires Arp2/3 complex-dependent actin assembly, and subsequently, myelin wrapping occurs at the innermost layer around the axon. In the CNS, these actions require actin disassembly mediated by MBP in OL (Bacon et al., 2007; Zuchero et al., 2015). Further, redistribution of F-actin to the leading edge of the inner tongue of the myelin sheath is essential for the wrapping process; thus, the growth of myelin is managed by actin depolymerizing factor/cofilin1 activity (Nawaz et al., 2015). To wrap the membrane during the development of myelin, a large amount of newly produced membranes and proteins are transported by the exocytotic vesicle transport system. At present, the mechanism for moving myelin membrane in its wrapping and exocytotic vesicle transport of myelin membranes and proteins has not yet been clarified. Myo1d may contribute to these two processes.

Previously, we reported that Myo1d is enriched in the outer and the inner cytoplasm-containing loops *in vivo* (Yamazaki et al., 2014). In particular, in this study, we demonstrated that Myo1d is localized to the leading edge of myelin-like membrane in cultured OL. Since the leading edge of myelin-like membrane in cultured OL corresponds with the inner tongue, Myo1d may engage in the wrapping process of myelin. Myosin I family members have pleckstrin homology-like domain which binds to phosphoinositides (Hokanson et al., 2006; Feeser et al., 2010; Komaba et al., 2010; Patino-Lopez et al., 2010). Phosphoinositides are molecules that can mediate various membrane dynamics (see reviews in Di Paolo and De Camilli, 2006; Balla, 2013; Marat and Haucke, 2016). Recently, it was reported that myelin wrapping proceeds directionally by a phosphatidyl inositol-3,4,5-triphosphate-dependent mechanism at the inner tongue (Snaidero et al., 2014). Since Myo1d also has a pleckstrin homology-like domain in its tail (Hokanson et al., 2006; Feeser et al., 2010; Komaba et al., 2010; Patino-Lopez et al., 2010), Myo1d may participate in myelin membrane dynamics mediated by phosphoinositides such as direct binding with the myelin membrane.

Myelin wrapping requires unidirectional management of the progress of myelination in order to form its multilamellar structure. In Drosophila, *situs inversus* is induced by a mutant Myo1d homolog (Myo31DF) during development (Hozumi et al., 2006; Speder et al., 2006). Myo31DF is involved in left–right asymmetric (chiral) movement of the plasma membrane in which it interacts with beta-catenin and DE-cadherin (Taniguchi et al., 2011; Petzoldt et al., 2012). Recent reports have revealed that the interaction between actin and myosin is related to the left–right asymmetric movement of the plasma membrane (Naganathan et al., 2014, Tee et al., 2015). Therefore, during myelin wrapping, Myo1d associated with F-actin may participate in the unidirectional movement of myelin membrane.

OLs express many SNARE proteins (Madison et al., 1999; Feldmann et al., 2009; Schardt et al., 2009) and Rab family members (Bouverat et al., 2000; Rodriguez-Gabin et al., 2004; Anitei et al., 2009) related to vesicle transport. VAMP3 and VAMP7 are involved in the recycle pathway of PLP transport (Feldmann et al., 2011). Rab3A participates in myelin membrane biogenesis. A large number of molecules have been linked to either the endocytic or recycling pathways or to the ER-Golgi network. However, the exocytic machinery in mature OL is still unclear. We demonstrated here that although Myo1d-positive signals were partially colocalized with PLP-positive signals, the loss of Myo1d caused an accumulation of PLP in the OL cell body. Therefore, in myelin formation, Myo1d may be involved in exocytic vesicle transport of myelin proteins, including PLP. The tail domain of Myo1d likely interacts with other molecules, but specific molecules remain to be identified. To elucidate the mechanism of Myo1d-involved exocytosis, the identification of the binding partners and cargo molecules may be required.

In relation to disease, Myo1d has been identified as a candidate gene for participation in autism spectrum disorders (Stone et al., 2007). Recently, the role of myelination in psychiatric disease such as schizophrenia, depression, bipolar disorder, and autism spectrum disorder has begun to be investigated (see reviews in Haroutunian et al., 2014; Long and Corfas, 2014; Roussos and Haroutunian, 2014; Toritsuka et al., 2015). Abnormalities of white matter have been detected in brains of patients with these disorders and expression of several myelin genes have been linked to these disease conditions. Canavan disease is a neurodegenerative disease caused by deficiency in aspartoacylase (ASPA; Hoshino and Kubota, 2014). In this disease, ASPA deficiency causes loss of OLs and other degenerative changes, including the appearance of spongy myelin in postnatal development (Mattan et al., 2010; see review in Hoshino and Kubota, 2014). Because Myo1d has been reported to interact with ASPA *in vitro* and colocalizes with ASPA in brain (Benesh et al., 2012), Myo1d may also be related to Canavan disease, a fatal neurological disorder that results in the deterioration of myelin. Thus, it is necessary to identify the functional consequences of the interaction between Myo1d and ASPA. Thus, the role of Myo1d in myelination may be important in mental health and neurodegenerative disease.

In conclusion, our data provide strong evidence that Myo1d is colocalized with F-actin at the leading edge of the myelin membrane and is crucial for myelin formation during later stages of OL differentiation. Research into the identification of the Myo1d interacting molecules are now proceeding.

## Summary

The knockdown using specific siRNA of unconventional myosin ID in OLs induces morphological changes and resulting apoptosis. Additionally, myosin ID-knockdown impairs PLP transport in OL. These suggest that myosin ID may contribute to myelin membrane dynamics.

## Supplementary Material

Supplementary material

## Supplementary Material

Supplementary material

## Supplementary Material

Supplementary material
