# Supplementary material for: Knockdown of Unconventional Myosin ID Expression Induced Morphological Change in Oligodendrocytes
Source: ASN Neuro. 2016 Sep 21;8(5):1759091416669609. doi: 10.1177/1759091416669609 (PMC5036140; doi:10.1177/1759091416669609)
Supplement: Supplementary material [file Supplemental_Figure.pdf]

Supplemental Figure

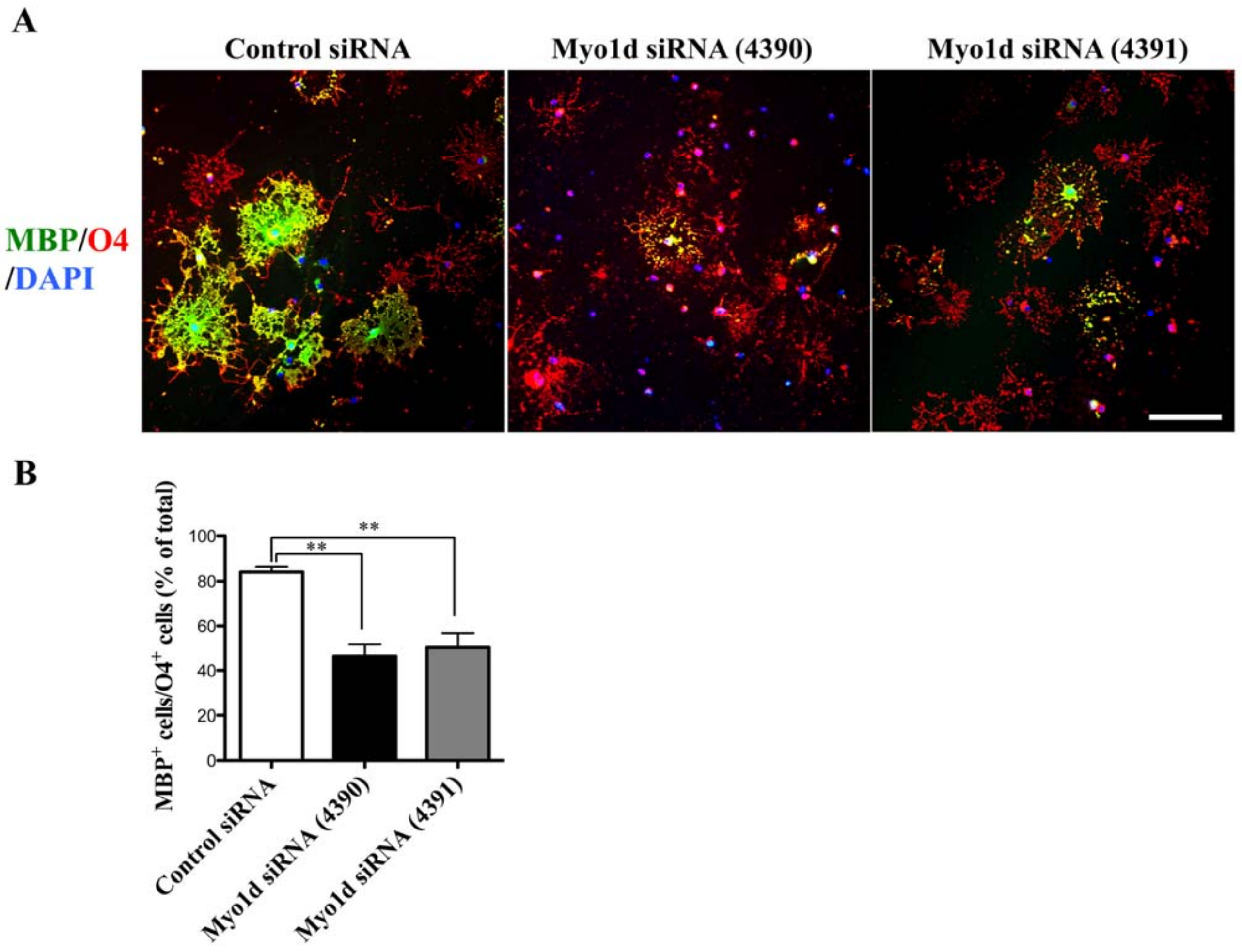

Supplemental figure. Decreases in the number of mature OL after knockdown of *Myo1d* expression using two other *Myo1d*-siRNA sets. (A) Cultured OLs were transfected with *Myo1d*- (4390 or 4391) or control-siRNA (100 nM each) for 48 hr beginning 3 days after differentiation. *Myo1d*-siRNA-transfected OLs were double-stained using anti-MBP (green) and anti-O4 antibodies (red). MBP-positive membrane sheets were almost disappeared by each *Myo1d*-siRNA transfection. Nuclei were counterstained with DAPI (blue) staining. Scale bar, 100 µm. (B) In quantitative analysis, the number of MBP-positive OLs was significantly decreased after *Myo1d* siRNA transfection (control siRNA, 84.12 ± 2.4%; Myo1d siRNA-4390, 46.49 ± 5.3%; Myo1d siRNA-4391, 50.38 ± 6.3%). Graph indicates the mean ± S.E.M. obtained from 5 fields in 2 independently-treated cover slips derived from 1 OL preparation (n=10). Asterisks indicate P<0.01 by Tukey-Kramer test after one-way ANOVA.

Supplemental information

The sequences of the double strand *Myo1d*-siRNA sets were as follows:

Myo1d-siRNA (4390)
Sence siRNA: 5'-CCAUGAUUGCUUUAGUAGATT-3'
Antisence siRNA: 5'-UCUACUAAAGCAAUCAUGGTT-3'

Myo1d-siRNA (4391)
Sence siRNA: 5'-CUAAUGAGCUGAAACGCAATT-3'
Antisence siRNA: 5'-UUGCGUUUCAGCUCAUUAGTT-3'
